# Multidrug-resistant *Mycobacterium tuberculosis* in HIV-Infected Persons, Peru

**DOI:** 10.3201/eid0912.020731

**Published:** 2003-12

**Authors:** Pablo E. Campos, Pedro G. Suarez, Jorge Sanchez, David Zavala, Jorge Arevalo, Eduardo Ticona, Charles M. Nolan, Thomas M. Hooton, King K. Holmes

**Affiliations:** * University of Washington, Seattle, Washington, USA; † Cayetano Heredia University, Lima, Peru; ‡ Peruvian Ministry of Health, Lima-Peru; § Asociacion Civil Impacta Salud y Educacion (IMPACTA), Lima, Peru; ¶ Public Health Seattle-King County, Seattle, Washington, USA

**Keywords:** Tuberculosis, multidrug resistance, HIV infection, Peru, isoniazid, rifampin

## Abstract

During 1999 to 2000, we identified HIV-infected persons with new episodes of tuberculosis (TB) at 10 hospitals in Lima-Peru and a random sample of other Lima residents with TB. Multidrug-resistant (MDR)-TB was documented in 35 (43%) of 81 HIV-positive patients and 38 (3.9%)of 965 patients who were HIV-negative or of unknown HIV status (p < 0.001). HIV-positive patients with MDR-TB were concentrated at three hospitals that treat the greatest numbers of HIV-infected persons with TB. Of patients with TB, those with HIV infection differed from those without known HIV infection in having more frequent prior exposure to clinical services and more frequent previous TB therapy or prophylaxis. However, MDR-TB in HIV-infected patients was not associated with previous TB therapy or prophylaxis. MDR-TB is an ongoing problem in HIV-infected persons receiving care in public hospitals in Lima and Callao; they represent sentinel cases for a potentially larger epidemic of nosocomial MDR-TB.

Multidrug-resistant tuberculosis (MDR-TB) threatens global TB control and has been identified in almost all surveyed countries. During 1994 to 1997, the World Health Organization (WHO)-International Union against Tuberculosis and Lung Disease identified high prevalences of MDR-TB in the former Soviet Union, Asia, the Dominican Republic, and Argentina (*1*).

MDR-TB has been associated with inadequate treatment regimens, poor adherence to treatment, poorly managed TB-control programs, and unenforced hospital infection control programs, as well as HIV infection (*2*). HIV infection influences the natural history of TB in several ways: active TB occurs within 6 months of acquiring *Mycobacterium tuberculosis* infection in 37% of persons with HIV-induced immunosuppression (*3*) but occurs in 2% of immunocompetent adults during the first year after acquiring *M. tuberculosis* infection (*4*). HIV-infected persons with TB also have increased frequency of disseminated and meningeal disease, other extrapulmonary diseases, atypical clinical signs and symptoms, drug-related adverse events, and negative sputum smears for acid-fast bacilli (*5*).

Nosocomial outbreaks of MDR-TB involving HIV-infected persons have occurred in the United States and other industrialized countries (*6*–*9*), but improved treatment and hospital infection control programs have contained these initial outbreaks of MDR-TB. However, such outbreaks are increasingly recognized in countries with more limited resources (*10*,*11*).

In Peru, the National TB Control Program received reports of 35,685 new TB cases in 1999 (incidence 141.4/100,000) (*12*). Passive reporting to the National TB Control Program showed HIV prevalences from 1% to 1.5% in TB patients during 1996 to 1999 (*12*), but prospective testing of 1,043 patients with a new event of TB in Lima and Callao from September to October 1999 found HIV seropositivity in 2.3% (P.E. Campos, unpub. data). Furthermore, TB was the AIDS-defining illness in 10,939 (28%) AIDS cases in Peru (*13*), and 50% of persons with AIDS in Peru develop TB at some point in their disease.

A National TB Control Program survey during 1999 found MDR-TB in 57 (3%) of 1,879 Peruvians with a first episode of TB and in 32 (12.3%) of 260 with previously treated TB (*14*). Of 2,101 TB patients in that survey, 8 had a previous diagnosis of HIV infection. In 1997, at the Dos de Mayo Hospital in Lima, which provides care to the largest number of HIV-infected persons, 9 (34.6%) of 26 HIV-infected patients with TB had MDR-TB (*15*).

Mortality rates during TB treatment of Peruvian HIV-infected patients has been high; 63%, 51%, 49%, and 39% of patients with a first episode of TB died during treatment that was started in 1996, 1997, 1998, and 1999, respectively (*12*,*16*). These rates contrast with a mortality rate of 9% in HIV-infected persons with pulmonary TB in Haiti (*17*). Treatment regimens prescribed for TB in developing countries are rarely based on susceptibility testing, and since 1987, HIV-infected patients with a first episode of TB in Peru have received a standard course of rifampin, isoniazid, pyrazinamide, and ethambutol in compliance with WHO recommendations (*18*). Therefore, MDR-TB might contribute to the high mortality rate for TB in HIV-infected Peruvians.

We performed this study to further characterize the prevalence and pattern of MDR-TB in HIV-infected adults in Lima and the adjacent port of Callao in Peru and to explore preventable risk factors that could be used to design, implement, and evaluate better preventive and therapeutic interventions.

## Methods

### Study Design and Population

This study was designed to evaluate the prevalence of MDR-TB in HIV-infected patients >18 years of age and to assess potential risk factors for MDR-TB in HIV-infected persons with TB. The study was carried out from February 1999 to January 2000 in Lima and Callao. Lima is the capital city of Peru, and Callao is the adjacent main port; together they have a population of 8,239,891, representing 32% of the national population. Lima and Callao accounted for 78% of all Peruvian AIDS cases reported from 1983 to September 2000 (*13*) and for 56% of all TB cases reported in 1999 (*12*). The research protocol for using human participants in this study has been reviewed and approved by the Human Subject Review Committee of the University of Washington and the Ethical Committee of the Scientific Research Office at the Cayetano Heredia University. Informed consent was obtained from the participants.

From February 1999 to January 2000, we introduced an active surveillance system to identify HIV-infected adults with a new event of TB, defined as a first episode of TB or a relapse, at the 10 public hospitals that provide care for most HIV-infected persons living in Lima and Callao. Each of these 10 hospitals has a unit of the National TB Control Program where every patient with a suspected or confirmed diagnosis of TB is referred for further work-up, treatment, follow-up, or referral. To identify HIV-infected patients with a new episode of TB, one trained interviewer periodically visited each of these units. In addition, we encouraged all clinicians to send clinical specimens for isolation of *M. tuberculosis*, and all laboratories to submit such isolates to TB control program reference laboratories for susceptibility testing.

MDR-TB was defined as resistance to both isoniazid and rifampin, with or without resistance to other drugs. Case-patients were adults with previously diagnosed HIV infection and with a new episode of TB, whose isolates of *M. tuberculosis* were MDR-TB. HIV-seropositive controls were adults with previously diagnosed HIV infection and a new event of TB, whose isolates of *M. tuberculosis* were susceptible to isoniazid or rifampin. HIV-positive status was defined by previous, repeatedly positive enzyme-linked immunosorbent assay (ELISA) confirmed by immunofluorescence or Western blot.

From February trough September 1999, a total of 972 adult patients with a new episode of smear-positive TB in Lima and Callao who received care at Ministry of Health facilities were included in the National TB Control Program’s surveillance of resistance to anti-TB drugs. Of these 972, a total of 116 had been previously tested for HIV: 7 (6%) were HIV seropositive. From the remaining 965 participants who were HIV seronegative or not tested for HIV infection, we randomly selected a second control group of 153 participants.

### Enrollment, Interview, and Treatment

Both trained interviewers periodically visited each of the 10 hospitals to identify, enroll, and interview HIV-infected patients with a new episode of TB. Because classification as “case” or “control” for HIV-infected patients was determined by drug susceptibility-test results, which became available after 3 or 4 months, each HIV-infected adult with a new episode of TB was eligible to participate, and interviewers were blinded as to susceptibility test results. One member of the local TB team introduced the interviewer, who explained the study and invited the patient to participate. Interviewers contacted adults with TB who were HIV seronegative or of unknown HIV status belonging to the HIV–seronegative control group at their homes, where they were invited to participate, enrolled, and interviewed. Patients giving written informed consent underwent a standardized face-to-face interview concerning demographic characteristics, past and current medical history, and potential exposures to *M. tuberculosis* during the 12 months before onset of TB symptoms. Healthcare workers at each facility followed national guidelines of the TB and the AIDS control programs in providing further treatment and follow up of study participants. Data collected were handled exclusively by the study team, ensuring confidentiality.

### Laboratory Methods

Primary isolations were attempted by using Lowenstein-Jensen or Ogawa medium at each hospital laboratory and at local reference laboratories serving the national surveillance study. Susceptibility testing was carried out at the Mycobacteria Laboratory of the Peruvian National Institute of Health, the national reference laboratory for susceptibility testing, or at one of three local mycobacterium reference laboratories. These laboratories used the proportion method to determine the sensitivity profile of each strain (*19*), using the following critical concentrations (μg/mL): isoniazid, 0.2; rifampin, 40; streptomycin, 4; ethambutol, 2; and pyrazinamide, 100. The National Reference Laboratory underwent external quality control by the Pan American Health Organization/WHO Instituto Panamericano de Protección de Alimentos y Zoonosis (INPPAZ) and performed quality control for the three local reference laboratories (*20*).

### Data Analyses

SPSS 10.0 software (SPSS, Inc., Chicago, IL) was used for data entry and analyses. Percentages of MDR-TB were compared in HIV-positive and HIV-negative patients with TB, and risk factors for MDR-TB in HIV-infected patients were explored by calculation of odds ratios (OR) for dichotomous variables. Medians of continuous variables were compared by using Student t test for independent samples.

## Results

### Characteristics of Patients with TB

Of 415 HIV-seropositive patients diagnosed and reported with a new episode of TB (on the basis of acid-fast smear or culture results or on clinical characteristics) at the 10 hospitals during the study period, 157 (38%) were not interviewed: 87 had already left the hospital and could not be located at referral health centers, 67 were in poor clinical condition and unable to answer the questionnaire, and 3 declined to participate. The remaining 258 seropositive participants interviewed averaged 32 years of age (range 18 to 62) and reported 9.8 years of education; 77% were men, 57% single, and 20% had had at least one previous episode of TB. Isolation of *M. tuberculosis* was attempted in 239 (93%); 135 were culture-positive, 61 were smear positive for acid-fast bacilli, and 62 were diagnosed on the basis of clinical criteria. Drug-susceptibility testing was requested for all positive cultures and completed on 81, who were the focus of subsequent analyses; the remaining strains were lost before or during transport to the National Reference Laboratory for susceptibility testing.

Of the 153 TB patients randomly selected as controls without known HIV infection, we located 110; 108 agreed to participate. They averaged 29.3 years of age (range 18 to 80) and reported 10.3 years of education; 57% were men, 52% single, and 9.3% had had at least one previous episode of TB.

Table 1 presents characteristics of participants with and without known HIV infection for whom drug-susceptibility testing was completed. Because HIV infection in Peru disproportionately affects men who have sex with men, HIV-seropositive persons were more often men and less often married, divorced, or widowed; family income and family ownership of the home were more common among those not known to have HIV infection. More importantly, previous histories of TB or TB prophylaxis were significantly more common in HIV-infected participants, as was history of contact with hospitals.

**Table 1:** Demographic characteristics, past medical history, and potential exposures to tuberculosis (TB) of participants with and without known HIV infection^a^

Variable	HIV-infected (n = 81)	Negative or unknown HIV status (n = 108)	p value or OR (9 5% CI)^b^
	Mean ± SD, or %	Mean ± SD, or %	
Age	31.6 ± 7.0	29.3 ± 11.9	0.13
Male	80	57	3.0 (1.6 to 5.9)
Marital status			
Single	59	52	0.42
Married	9	20	0.05
Divorced/widowed	11	28	<0.01
Cohabitant	21	0	<0.01
Y of education	9.6 ± 2.8	10.3 ± 2.1	0.08
No. residents/no. bedrooms	3.0 ± 2.0	2.8 ± 1.4	0.48
Family income	603 ± 333	845 ± 404	<0.01
Family owns home	75	88	0.4 (0.2 to 0.9)
Employed^d^	63	28	4.4 (2.4 to 8.2)
Exposure to TB at work^d^	17	3	7.3 (2.0 to 26.4)
Exposure to TB at home^d^	16	1	20 (2.6 to 160)
Previously treated TB^d^	27	9	3.6 (1.6 to 8.3)
Ambulatory care at health centers^c^	21	5	5.5 (1.9 to 16)
Ambulatory care at hospitals^c^	40	1	70 (9.3 to 526)
Inpatient care^c^	14	0	<0.001
Inpatient MoH hospitals^c^	11	0	<0.001
Any exposure to hospital^c^	44	1	86 (11.4 to 644)
TB prophylaxis^e^	22	0	<0.001

^a^OR, odds ratio; CI, confidence interval; MoH, Ministry of Health. ^b^p values proved when comparing means or when one column had a value of 0. ^c^Variables reported by patients to have occurred during the 12 months before onset of symptoms. ^d^Exposure to someone with TB at work or at home during the 12 months before onset of symptoms. ^e^Ever in the past.

### Drug-Susceptibility Test Results

The prevalence of MDR-TB was 43% in the 81 HIV-seropositive patients with available susceptibility test results and 3.9% in the 965 patients whose HIV status was negative or unknown (p < 0.001); only 1 (0.9%) of 108 patients whom we randomly selected and interviewed from this group of 965 TB patients had an MDR-TB isolate (Table 2). The prevalence of resistance to any drug was also higher in *M. tuberculosis* isolates from HIV-positive patients than in isolates from the 965 without known HIV infection.

**Table 2:** Drug-susceptibility profiles of *Mycobacterium tuberculosis* isolates from participants with and without known HIV infection^a^

Characteristic	HIV positive	HIV negative or unknown status	
	Patients	Interviewed patients	All patients
	N (%)	N (%)	N (%)
Total no. tested for drug resistance	81 (100)	108 (100)	965 (100)
Fully susceptible isolates	28 (35)	92 (85)	789 (82)
Any resistance	52 (65)	16 (15)	76 (18)
Any H resistance	42 (52)	11 (10)	110 (10)
Any R resistance	39 (48)	2 (2)	53 (6)
Any E resistance	19 (24)	0 (0)	27 (3)
Any S resistance	39 (48)	12 (11)	88 (9)
Any Z resistance	28 (35)	NA	NA
Multidrug resistance	35 (43)	1 (1)	38 (3.9)
Only HR resistance	3 (4)	NA	NA
HRZ resistance	25 (32)	NA	NA
HRESZ resistance	11 (14)	NA	NA

^a^NA, not available; H, isoniazid; R, rifampin; E, ethambutol; S, streptomycin; Z, pyrazinamide; HRZ resistance includes any strain resistant at least to H and R and Z.

Drug-susceptibility test results were available for 26 (16.6%) of the 157 HIV-seropositive patients we did not interview; 35% of these patients had MDR-TB, not significantly different from the 43% of those we did interview (p = 0.44).

### HIV Patients with MDR-TB and with Non–MDR-TB

Of HIV-seropositive participants with TB (Table 3), MDR-TB was significantly associated with TB diagnosed at hospital A (OR 3.7, 95% confidence interval [CI] 1.3 to 10), with employment during the 12 months before the onset of symptoms, and with exposure to TB at work, but not with age, sex, marital status, education level, crowding in the home, or low income. MDR-TB was not significantly associated with previous episodes of TB or with TB prophylaxis; the proportion who had received TB prophylaxis or had a previous episode of treatment for TB was 17 (49%) of 35 with MDR-TB versus 20 (43%) of 46 without MDR-TB (OR 1.2, 95% CI 0.5 to 3.0).

**Table 3:** Demographic characteristics, past medical history, and potential exposures to TB of HIV-infected participants with MDR-TB and without MDR-TB^a^

Variable	HIV-infected with MDR-TB (n = 35)	HIV-infected without MDR-TB (n = 46)	OR (95% CI)	p value	
	Mean ± SD or %	Mean ± SD or %			
Employed^b^	46	76		0.3 (0.1 to 0.7)	
Exposure to TB at work^c^	6	26		0.2 (0.04 to 0.8)	
Exposure to TB at home^c^	20	13		1.7 (0.5 to 5.5)	
Previously treated TB^d^	29	26		1.1 (0.4 to 3.0)	
Duration of symptoms	6.7 ± 5.4	7.8 ± 6.6	0.44		
Ambulatory care at health centers^b^	14	26		0.5 (0.2 to 1.5)	
Ambulatory care at hospitals^b^	43	37		1.3 (0.5 to 3.1)	
Ambulatory care at hospitals^e^	3924 ± 5749	2737 ± 3361	0.48		
Inpatient care^b^	17	11		1.7 (0.5 to 6.1)	
Inpatient MoH hospitals^b^	11	11		1.1 (0.3 to 4.3)	
Days of hospitalization^b^	18 ± 11	30 ± 26	0.36		
Diagnosed at hospital “A”	80	52		3.7 (1.3 to 10)	
Any exposure to hospital^b^	51	39		1.6 (0.7 to 4.0)	
HIV support group^b^	11	7		1.9 (0.4 to 8.9)	
TB prophylaxis^d^	26	20		1.4 (0.5 to 4.1)	
Mo. TB prophylaxis	6.7 ± 5	4.4 ± 3.3	0.27		
PCP prophylaxis^d^	57	44		1.7 (0.7 to 4.2)	
Extrapulmonary TB	29	44		0.5 (0.2 to 1.3)	

^a^MDR-TB, multidrug-resistant tuberculosis; OR, odds ratio; CI, confidence interval; MoH, Ministry of Health; PCP, *Pneumocystis carinii* pneumonia. ^b^Variables reported by patients to have occurred 12 months before onset of symptoms. ^c^Exposure to someone with TB at work or at home 12 months before onset of symptoms. ^d^Ever in the past. ^e^As a continuous variable: total minutes of exposure 12 months before onset of symptoms.

### Hospital Characteristics

Table 4 lists the 10 major public hospitals in Lima and Callao by number of persons with newly diagnosed AIDS reported during 1999, number with TB diagnosed during 1999, number of HIV-infected patients with a new event of TB during the study, and percentage of isolates of *M. tuberculosis* identified as MDR-TB. All but one of the MDR-TB cases were identified in the three hospitals reporting the largest number of HIV-seropositive patients with a new event of TB during 1999.

**Table 4:** Ranking of hospitals by number of newly diagnosed cases of AIDS, tuberculosis (TB), HIV, and TB co-infection, February 1999–January 2000^a,b^

Hospital	No. AIDS patients reported during 1999^c^	No. TB patients diagnosed during 1999	Total no. HIV-infected patients with a new diagnosis of TB	No. (%) HIV-infected patients with a new diagnosis of TB interviewed	No. MDR-TB ÷ total drug susceptibility test results available (%)	
A	154	1,985	204	116 (57)	28/52 (54)	
B	60	723	49	40 (82)	4/9 (44)	
C	139	1,068	41	24 (59)	2/7 (29)	
D	10	891	37	29 (78)	0/4 (0)	
E	33	635	33	21 (64)	0/5 (0)	
F	95	608	29	11 (38)	0/1 (0)	
G	9	425	12	11 (92)	0/1 (0)	
H	30	169	6	4 (67)	0/1 (0)	
I	4	130	3	1 (33)	0/0 (NA)	
J	2	131	1	1 (100)	1/1 (100)	
Total	554	6,765	415	258	35/81 (43)	

^a^Among those co-infected with TB and HIV, the number (%) with MDR-TB. ^b^MDR-TB; multidrug-resistant tuberculosis. ^c^Number of AIDS cases reported to the National AIDS and STD Control Program.

### Assessment of Possible Bias

Interviewed patients with (N = 81) or without (N = 177) susceptibility results were similar with respect to age, years of education, family income, symptom duration, time since previous TB, TB prophylaxis in the past, crowding in the home, gender, marital status, home ownership, and employment; and during the past 12 months, exposure to TB at work, exposure at home, ambulatory care at health centers, ambulatory care at hospitals, inpatient hospital care, and participation in HIV or TB support groups. History of previous TB was somewhat more frequent in those tested (27%) than in those not tested (17%, p = 0.09), and history of *Pneumocystis carinii* pneumonia (PCP) prophylaxis was more frequent among those tested (50%) than in those not tested (32%, p < 0.01).

## Discussion

This study documents high rates of MDR-TB in HIV-infected persons with TB receiving care at public hospitals in Lima and Callao; MDR-TB was 11 times more common in these patients than in 965 TB patients without known HIV infection in Lima and Callao. In comparison to persons having TB without known HIV infection, the HIV-infected patients with TB had higher frequency of contact with health centers and hospitals, and MDR-TB was found mainly in patients receiving care at the three hospitals serving the largest number of HIV-infected patients with TB. Although participants with HIV infection more often had had previous episodes of TB, and presumably, only the HIV-infected persons had received TB prophylaxis, these factors were not significantly associated with MDR-TB in the HIV-infected patients with TB.

The 43% prevalence of MDR-TB among HIV-infected persons with TB found in this study is higher than the 36% previously found in Italy (*21*), and the 28.3% found in Argentina, both during epidemics of nosocomial MDR-TB (*1*).

Because early clinical manifestations of TB often develop in HIV-infected persons (*3*,*5*), they mark sentinel cases that first indicate outbreaks of nosocomial transmission of TB and may represent a larger number of nosocomial transmission of TB.

Three factors drive TB transmission: the rate of exposure of susceptible to infectious persons, the efficiency of transmission per exposure, and the average duration of infectiousness once infection has occurred (*22*). For HIV-infected persons, higher exposure to TB in general could easily result from routine periodic visits to clinical settings also frequented by patients with TB (as occurs in Lima and Callao, where most persons with HIV infection attend HIV/AIDS clinics located at large hospitals); from the mixing of HIV-infected patients with TB patients in these settings because of the archaic practice of mixing patients who have communicable diseases (like TB and HIV infection) in large multiple-bed wards. MDR-TB case-patients tend to be selectively hospitalized because they do not respond to routine therapy.

Conditions that increase exposure to TB, such as overcrowding, long waiting times in clinics, sharing of facilities, and large open multiple-bed wards, are also common in medical institutions in the developing world. Increased efficiency of transmission per exposure could occur if HIV-related immunosuppression increases host susceptibility to acquisition of infection by *M. tuberculosis* in general or by MDR-TB strains of TB in particular.

Several factors could prolong the duration of infectiousness of MDR-TB in HIV-immunosuppressed persons, including continued use of isoniazid- and rifampin-based regimens as initial therapy for TB in HIV-infected persons, even during ongoing outbreaks of MDR-TB transmission. Patients receiving ineffective treatment will not improve and will more often use clinical services as outpatients and as inpatients, increasing the exposure to MDR-TB of other patients. In addition, increased frequency of atypical clinical pictures and smear-negative results, relatively common features in HIV-infected patients with TB, can contribute to delayed diagnosis and prolonged infectivity.

Evidence suggests that HIV infection favors the emergence of acquired drug resistance in individual patients during treatment (*23*,*24*). HIV-infected persons have a higher risk for acquisition of isolated rifampin resistance (*25*,*26*), and once- or twice-weekly rifamycin-based regimens increase the risk for acquired rifamycin resistance in TB patients with advanced HIV disease (*27*–*29*). MDR-TB is more often a consequence of addition of rifampin resistance than of addition of isoniazid resistance (*22*).

Limitations of this study included the fact that TB cases with HIV infection were recruited from hospitals where patients with known HIV infection receive care in Lima, whereas HIV-negative controls were recruited from nonhospital clinics. However, methodologic features and results of this evaluation suggest that results may be generalizable to all HIV-infected patients with TB receiving care within the Ministry of Health system in Lima and Callao. Of 457 new episodes of TB in HIV-infected persons >18 years in Lima and Callao during 1999 (*12*), we identified 415 cases (91%) through the TB units at each of these 10 hospitals during the 12-month study. We systematically interviewed HIV-infected patients well in advance of knowing whether they had MDR-TB, and we systematically interviewed a random sample of TB patients without known HIV infection from a larger representative sample of TB patients from Lima and Callao. Participants with known HIV infection were selected consecutively and patients without known HIV infection were selected randomly from all case-patients of TB receiving care at public health services. Comparisons of those interviewed versus not interviewed, and those tested and not tested for drug susceptibility suggested no bias, except that those tested had somewhat higher frequencies of previous TB and of PCP prophylaxis. The latter could reflect greater immunosuppression or greater contact with the medical care system. Although both could have biased results toward higher estimates of MDR-TB prevalence among HIV-seropositive patients, a previous history of TB or of TB prophylaxis was not associated with MDR-TB.

We were unable to interview all HIV-positive patients with TB diagnosed during the study, raising the question of whether the prevalence of MDR-TB was higher in those we interviewed than in those not interviewed. However, the 43% frequency of MDR-TB in those we interviewed did not differ significantly from the 35% prevalence of MDR-TB in the 26 patients who we did not interview for whom susceptibility testing was performed. Finally, concerning the fact that we did not obtain HIV serologic finding from all of the 108 controls with negative or unknown HIV status, if the prevalence of HIV had been higher than the 2.3% prevalence observed during prospective testing in Lima in 1999, such misclassification bias would only have reduced the differences observed between groups.

Community-based studies throughout the United States have documented increased prevalence of MDR-TB in HIV-infected people (*2*), and in a recent report of several surveys (*30*), MDR-TB was found more often in HIV-infected patients than in HIV-uninfected patients, but after adjustment for previous treatment for TB the difference between HIV-infected and HIV-uninfected patients was no longer statistically significance. However, in our survey, even after eliminating patients with history of prior episodes of TB or TB prophylaxis, MDR-TB was still seen in 18 (43%) of 44 isolates from HIV-infected persons versus 24 (3%) of 814 HIV-negative controls (p < 0.001).

## Conclusions

Although rigorous compliance with infection control recommendations (*31*), particularly those related to engineering control, is difficult in developing countries, the combined epidemics of TB, MDR-TB, and HIV/AIDS make infection control measures essential. WHO guidelines recommend a hierarchy of controls (administrative, environmental, and personal respiratory protection), many of which entail little or no cost (*32*). Minimal measures must include strict respiratory isolation for patients with confirmed or suspected TB and mandatory wearing of appropriate masks for persons entering all patient rooms, for patients leaving their rooms when unavoidable, and for patients with cough when seen in clinics. These essential measures to avert TB transmission in healthcare settings have been demonstrated (*33*). Segregating persons with TB from those with HIV in individual rooms with negative air flow, establishing safer sputum sampling collection procedures, improving the laboratory support for early identification of TB and of MDR-TB, and providing more effective treatment regimens to patients at increased risk for MDR-TB are necessary. HIV testing of patients with TB and susceptibility testing of *M. tuberculosis* isolates from HIV-infected patients should be routine in settings where outbreaks or endemic transmission of MDR-TB is occurring in HIV-infected patients.

Nosocomial MDR-TB transmission at hospital A has been ongoing since 1997. Recently published IS6110 restriction fragment length polymorphism analysis of *M. tuberculosis* strains collected between July 1997 and April 1999 and belonging to HIV-positive inpatients clearly implicate nosocomial transmission of MDR-TB in this hospital (*34*). Similarly, outbreaks of nosocomial MDR-TB in HIV-infected persons have emerged first in New York, Buenos Aires, and Lima (settings providing hospital-based care, including antiretroviral therapy, for HIV-infected persons). Unrecognized MDR-TB outbreaks in other developing countries are likely. As delivery of antiretroviral therapy for HIV in developing countries proceeds, nosocomial exposure of HIV-infected persons to TB must be minimized. More effective surveillance, prevention, and treatment for MDR-TB are essential.
